# Prolonged Maternal and Child Health, Food and Nutrition Problems after the Kumamoto Earthquake: Semantic Network Analysis of Interviews with Dietitians

**DOI:** 10.3390/ijerph18052309

**Published:** 2021-02-26

**Authors:** Nobuyo Tsuboyama-Kasaoka, Mari Hamada, Kae Ohnishi, Sakiko Ueda, Yukako Ito, Hisae Nakatani, Noriko Sudo, Ritsuna Noguchi

**Affiliations:** 1 Section of Global Disaster Nutrition, National Institutes of Biomedical Innovation, Health and Nutrition, Tokyo 162-8636, Japan; marihama.mh@gmail.com (M.H.); skueda@nibiohn.go.jp (S.U.); 2 Japan Dietetic Association-Disaster Assistance Team, The Japan Dietetic Association, Tokyo 105-0004, Japan; d161894@hiroshima-u.ac.jp (Y.I.); sudo.noriko@ocha.ac.jp (N.S.); r.noguchi@thu.ac.jp (R.N.); 3 Faculty of Health and Medical Science, Teikyo Heisei University, Tokyo 170-8445, Japan; 117A51012@edu.thu.ac.jp; 4 Graduate School of Biomedical and Health Sciences, Hiroshima University, Hiroshima 734-8551, Japan; hinakata@hiroshima-u.ac.jp; 5 Natural Science Division, Faculty of Core Research, Ochanomizu University, Tokyo 112-8610, Japan

**Keywords:** disaster, child, infant, mother, nutrition, health

## Abstract

Infants need sufficient nutrients even during disasters. Only qualitative descriptive analysis has been reported regarding nutritional problems of mothers and children after the Kumamoto earthquake, and non-subjective analysis is required. This study examined issues concerning maternal and child health, food and nutrition after the Kumamoto earthquake using automatic computer quantitative analysis from focus group interviews (FGIs). Study participants (*n* = 13) consisted of dietitians in charge of nutrition assistance of infants in affected areas. The content of the interviews was converted into text, nouns were extracted, and co-occurrence network diagram analysis was performed. In the severely damaged area, there were hygienic problems not only in the acute phase but also in the mid-to-long-term phase. “Allergy” was extracted in the surrounding area in the acute and the mid-to-long-term phase, but not in the severely damaged area as the acute phase issue. In the surrounding area, problems have shifted to health and the quality of diet in the mid-to-long-term phase. This objective analysis suggested that dietary problems for mothers and children after disaster occurred also in the mid-to-long-term phase. It will be necessary to combine the overall trends obtained in this study with the results of qualitative descriptive analysis.

## 1. Introduction

After a natural disaster, infants, pregnant and lactating women need sufficient nutrients to live and need priority support [1]. However, support for mothers and children after a natural disaster is typically insufficient. It has been reported that even nursery school dietitians were not prepared to create alternative menus to provide emergency meals when food distribution was disrupted [2]. In fact, the survivors who had the most dietary problems after the disaster were infants. A survey of emergency shelters conducted about one month after the Great East Japan Earthquake (2011) showed that among survivors that needed nutrition assistance, the most prevalent were “infants who needed infant formula and baby food” [3]. After a natural disaster, there is a frequent shortage of special diets. In the survey regarding necessary support to the affected areas after the Great East Japan Earthquake, the “goods” category was the most needed [4]. Food was the most needed in the “goods” category, followed by gasoline, and thirdly, special diets. Special diets included infant formula and baby food.

In addition to such special diets, the meals provided to all survivors at emergency shelters are also inadequate. It was also reported that the nutritional status of survivors in the disaster area one month following the disaster was insufficient in both dietary quantity and quality, and that the meals during this period was carbohydrate-rich [3,5]. When there was an overall food inadequacy in emergency shelters, it is not possible to provide adequate nutrition assistance to infants who required special dietary consideration. Moreover, although stockpiling food is essential for protecting infants after a disaster, there is a limit to how much the local government can stock for vulnerable populations. According to a 2018 survey of municipalities nationwide in Japan, 35.3% of local governments stockpiled infant formula and 21.7% stored allergen-free foods [6]. The stockpile rate of special diet is increasing compared to a survey conducted in 2013 [7], but it is not sufficient.

Most of the reports were not dealing with detail and specific dietary problems especially for mothers, and not focused on the mid-to-long-term phase as time has passed since the Kumamoto earthquake and until now, three years later. Therefore, in 2020 we reported a qualitative descriptive analysis using focus group interview (FGI) data, regarding not only the acute phase but also mid- to-long-term phase to search what kind of health, food, and nutrition problems there were for mothers and children after the Kumamoto earthquake [8]. When the Kumamoto earthquake occurred on 14 April 2016, affected areas measured a maximum seismic intensity of 7 [9]. At its peak, the number of evacuees reached about 184,000 people on 17 April, and more than 850 emergency shelters were built. With the reopening of schools about one month after the foreshock (9 May), emergency shelters were significantly consolidated and all shelters were eventually closed seven months later, in November of the same year. In our previous qualitative and descriptive analysis [8], “importance of usual diet”, “difficult to stay in a shelter”, “difficulty in nutrition assistance”, and “insufficient food stockpile” were extracted as acute phase problems for mothers and children in the severely damaged area. In the mid-to-long-term phase, “systems and stockpiles replanning”, and “need a healthy and safe diet” were extracted in the severely damaged area. Contrary in the surrounding area, “cruel shelter environment”, “need special nutritional assistance”, “nutrition assistance system problem”, and “insufficient food stockpile” were extracted as acute phase problems. Furthermore, “anxiety about children’s health: obesity and low appetite”, “necessity of creating a nutritional assistance system”, and “need to continue stockpiling suitable for mothers and children” were extracted as the mid-to-long-term phase problems in the surrounding area.

However, these subjective and qualitative descriptive analysis did not sufficiently elucidate the depth of relevance between each issue. Furthermore, it cannot be denied that the analysts’ thoughts were subjective and there was a possibility of biased interpretations. In addition, it is not clear how changes in food supply systems and dietary habits will influence physical condition or appetite. Therefore, it is necessary to objectively analyze the problems and actual conditions of the health, food, and nutrition of infants, especially, and their mothers by a non-subjective method, and to elucidate the overall tendency from overlooking.

The purpose of this study was to clarify the health conditions, food and nutrition issues for children and their mothers during the acute to mid-to-long-term phases of the Kumamoto earthquake from objective and multiple perspectives analyzed by automatic computer quantitative analysis by using the same text data as from FGIs [8].

## 2. Materials and Methods

### 2.1. Participants

This study was conducted from FGIs to collect information the health conditions, food, and nutrition issues for children and their mothers during the acute to mid-to-long-term phase of the Kumamoto earthquake. Participants were registered dieticians and dietitians who worked in local government and nursery schools and were involved in nutrition assistance activities in areas affected by the Kumamoto earthquake. A focus was primarily placed on local government and nursery school dietitians who were mainly in charge of supporting infants and their mothers. To examine the effects of differences of damage, the areas where the damage by the earthquake was the most severe and the surrounding areas were selected. Recruitment was conducted using personal connections and snowball sampling, resulting in 13 applications being submitted. The participant number for a focus group interview generally suggested as being manageable is between 6 and 10 participants [10]. Therefore, the sample size was set to about 5 to 8 people per focus group to foster lively and free discussions.

Consent to participate in the study was obtained from the 13 dietitians, all of whom were females. Of the 13 interviewees, everyone participated, and no one dropped out of the study. Table 1 shows information about the participants from each group.

Focus group interviews were conducted with two groups. Group 1 consisted of dietitians who worked for the local government (*n* = 4) and in nursery schools (*n* = 1) in areas that suffered extensive damage during the earthquake (*n* = 5 total). Group 2 consisted of 8 local government dietitians who worked in the surrounding area that was less impacted by the disaster.

### 2.2. Data Collection Methods

Focus group methodology was used to explore the health conditions, food, and nutrition issues for children and their mothers from dietitian experience. From November to December 2019, semi-structured focus group interviews were conducted. Researcher with registered dietitian qualifications conducted the interviews as moderator. The moderator was trained and experienced in qualitative data collection methods including focus groups. All focus groups were assigned to the same moderator. The moderator developed the interview script using guidelines [10] and used a semi-structured interview script to help guide the focus groups (Appendix A), and the interview questions were asked using the same wording. Two different researchers (M.H. and Y.I.) reviewed the interview script for clarity and appropriateness. The time allotted for the interviews was approximately 2 h. The content of the interviews was as follows: (1) what was prepared for children before the disaster; (2) problems related to maternal and child diet, nutrition, and health that occurred in the acute phase after the disaster, and how they were handled; (3) problems related to maternal and child diet, nutrition, and health that occurred mid-to-long-term phase after the disaster and how they were handled; and (4) current problems related to maternal and child diet, nutrition and health. We defined the mid-to-long-term phase as when the turmoil has subsided, a phase of time since the disaster. Although it is a very rough classification, it was assumed that the acute phase was approximately a month immediately after the disaster, and the mid- to long-term period was a month or later in this interview about the Kumamoto earthquake. Questions were asked about the above 4 topics and free discussion was encouraged. Interviewees were asked to speak broadly, not only about maternal and child related issues they encountered at work, but also about what they had heard from others, including evacuees.

The moderator reviewed the study procedures and the informed consent form at the beginning of interview. We have obtained written consent from all participant whose comments were analyzed in this study. With the consent of the participants, the interviews were recorded using three IC recorders and transcriptions of the audio recordings were created by a specialty contractor. A note taker was present. FGIs were conducted until data saturation was reached [11]. Any identifying information such as personal or facility names were not used in the transcript. Additionally, the content of what was said during the interview was made anonymous and personal identification codes were not included.

### 2.3. Data Analysis Methods

The audio recordings of the FGIs were transcribed verbatim for each group and names and other personal identifiers were removed. Among the content of each topic (1 through 4), we used 3 topics except topic 1 (before the disaster) for this analysis. Topic 2 (acute phase after the disaster) was analyzed alone as the acute phase of the disaster. Topic 3 (mid-to-long-term phase after the disaster) and Topic 4 (current situation) were combined and analyzed together as the mid-to-long-term phase of the disaster, because there were no differences in the data between these topics.

To grasp the overall tendency by non-subjective method, we have used computer-assisted text analysis. Quantitative semantic network analysis is a method of organizing data in a text format and the content of these data is then analyzed. This analysis method can help the data analyst to avoid combining data in arbitrary ways. The aim of this analysis is to analyze the co-occurrence of the morphemes (the smallest meaningful unit of language) extracted and categorized from the interview data, and objectively express the child and maternal health, food and nutrition by the damaged level and the phases after disaster. The verbatim recordings text data were cleaned, and the moderator’s remarks, missing data, and notations unable to be read by the personal computer were excluded from text data. Furthermore, typographical errors, abbreviations, and abbreviated notations were unified, and fluctuations in notations were corrected by replacement work. After cleaning, the verbatim data were converted to the analysis text data regarding the acute phase and mid-to-long-term phase of the earthquake. Transcribed analysis text was first subjected to quantitative text mining analysis using KH Coder, a free software program developed by K. Higuchi at Ritsumeikan University in Japan (Available for download at https://sourceforge.net/projects/khc/, accessed on 27 April 2020) [12]. KH coder consists of R programming language, with the “ChaSen” language morphology-analysis system as the backend program and can analyze Japanese, English, German, Italian, Portuguese, and Spanish text [12]. The interview text data for analysis from two groups were served for sequential quantitative analysis. All text data were automatically decomposed into morphemes, then nouns were extracted. Finally, the interrelationships among nouns were visualized as the co-occurrence networks. In order to categorize the nouns, assisted by the “co-occurrence network mode”, we then visualized the spatial distribution of each noun in two dimensions by using the “multidimensional analysis mode”. All interview text data were analyzed in Japanese, and the results of co-occurrence network diagram were translated into English.

### 2.4. Ethical Considerations

The purpose of the interview was explained in writing in advance of the interview, and then an explanation was given orally before the start of the interview. Written informed consent was obtained from all participants involved in the study. This study was approved by the institutional ethics committees of the National Institutes of Biomedical Innovation, Health and Nutrition (approval no. KENEI-112).

## 3. Results

### 3.1. Co-Occurrence Network

**Figure 1:**
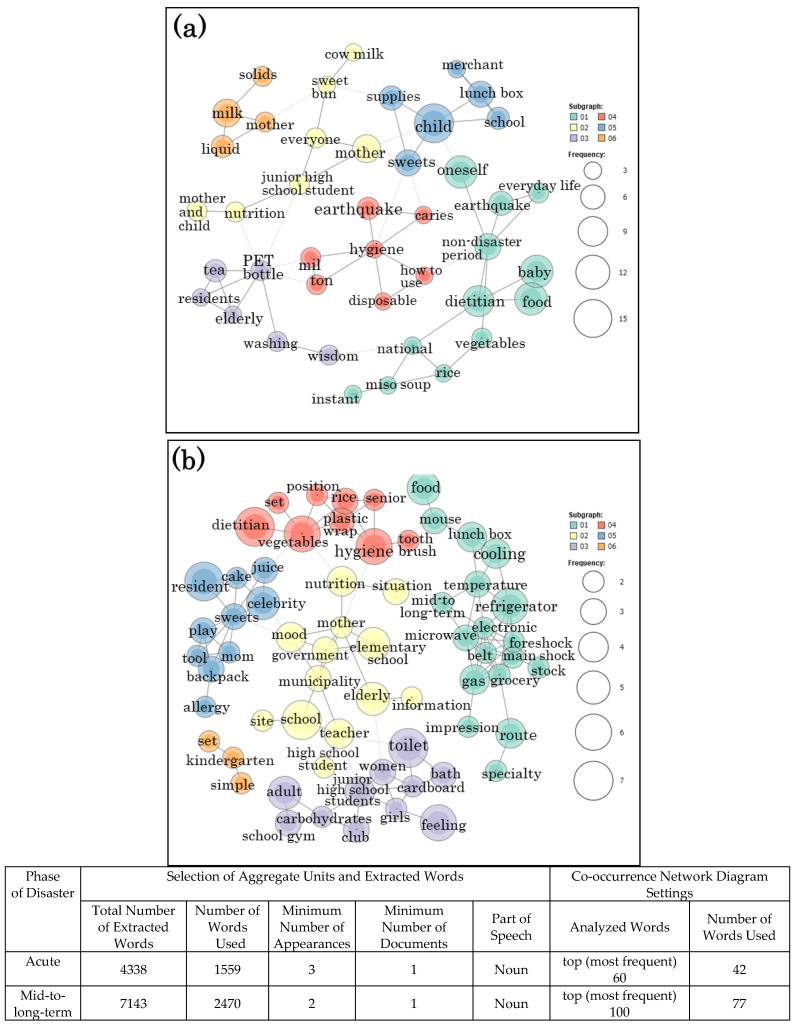
Co-occurrence network diagrams of Group 1 (severely damaged area) created using quantitative text analysis: Kumamoto earthquake. (**a**) acute phase; (**b**) mid-to-long-term phase.

**Figure 2:**
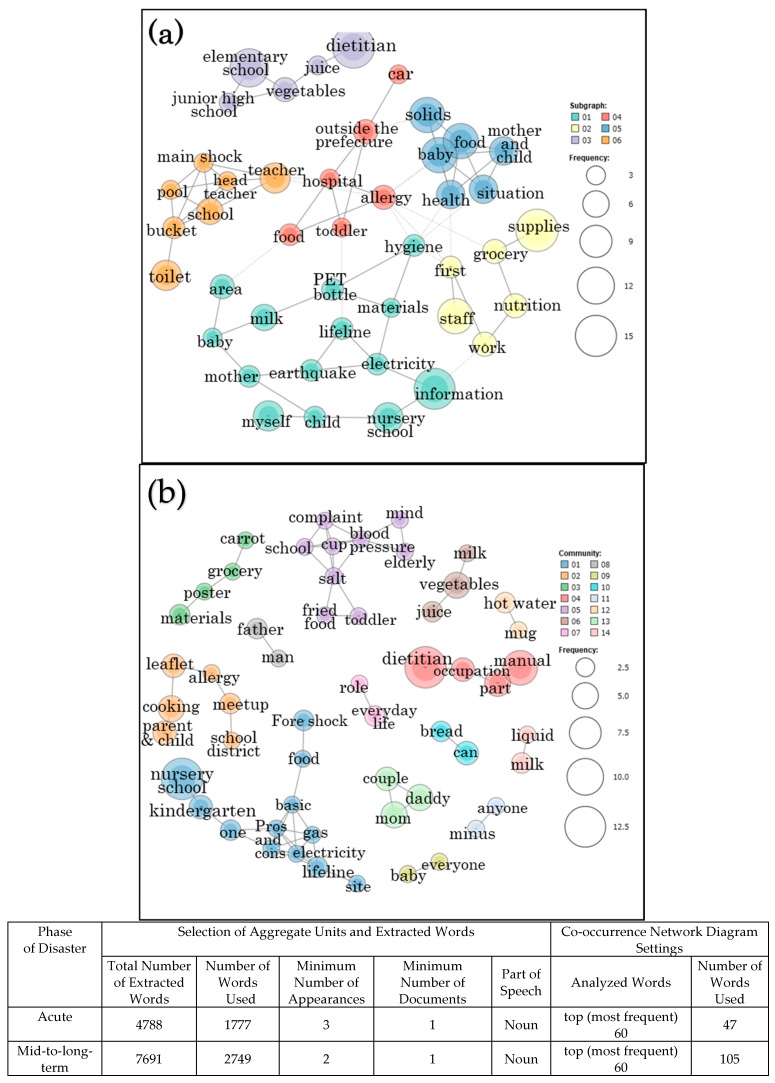
Co-occurrence network diagrams of Group 2 (Surrounding Area) created using quantitative text analysis: Kumamoto earthquake. (**a**) acute phase; (**b**) mid-to-long-term phase.

A co-occurrence network diagram was drawn for the purpose of summarizing the frequency of appearance of words and the relationship between extracted words. The co-occurrence network is a network diagram showing the strength of co-occurrence between words with similar appearance patterns, i.e., extracted words. The size of the circle indicates the frequency of words, and the distance of the lines connecting the circles indicates the depth of relevance. The results of the co-occurrence network diagram created using quantitative text analysis are presented in Figure 1 and Figure 2. Co-occurrence indicates that certain words in the text data appeared together, and a co-occurrence network is formed when these co-occurring words are connected using lines. Even if the expressions are different, if there are any connections in the transcript, then they are connected by a line. Compared to those connected by dotted lines, words connected by a solid line are relatively more related to each other.

### 3.2. Co-Occurrence Network Diagram of Maternal and Child Health, Food and Nutrition Problems in the Severely Damaged Area

Figure 1 shows the co-occurrence network diagrams of maternal and child health, food, and nutrition problems in group 1 as a severely damaged area after Kumamoto earthquake. The size and the number of occurrences of the extracted words are shown on the right side in Figures. Six subgraphs were shown in the co-occurrence network diagram of acute phase problem (Figure 1a). In the 05 subgraph containing the extracted words that appear frequently, the keywords “child”, “sweets”, and “lunch box” are included from the one with the largest circle. Similarly, looking at the connection of the extracted words for each group, the keywords were “dietitian”, “baby food” and “non-disaster period” in the 01 subgraphs, “milk” and “liquid” in the 06 subgraph, and “PET bottle” and “tea” in the 03 subgraph. From the connection of these keywords, it was possible to summarize that the food and beverages required for infants have become a problem. In the 04 subgraph, “hygiene”, “disposable” and “milton (antiseptic solution)” were summarized as their problem.

In the mid-to long-term phase, six subgraphs were indicated in the co-occurrence network diagram of problem as shown in Figure 1b. The 04 subgraph contained “hygiene”, “tooth brush”, and “vegetables”. “Toilet”, “bath” and “women” in the 03 subgraph, and “refrigerator”, “cooling” in the 01 subgraph were included. These results suggest that hygiene problems were prolonged as a factor related to the diet of mothers and children in severely damaged area.

### 3.3. Co-Occurrence Network Diagram of Maternal and Child Health, Food and Nutrition Problems in the Surrounding Area

Figure 2 shows the co-occurrence network diagrams of maternal and child health, food, and nutrition problems in group 2 from a surrounding area after Kumamoto earthquake. In the acute phase, six subgraphs were indicated in the co-occurrence network diagram of problems as shown in Figure 2a. As shown in the 05 subgraph, “solids” and “baby food” were also extracted as the problems even in the surrounding area, but “allergies”, “hospital” and “foods”, etc. were extracted in relation to them, and more qualitative problems. In addition, “toilet”, “buckets”, and “pool”, etc. were extracted in the 06 subgraph, and “hygiene”, “lifeline”, “electricity” and “PET bottle”, etc. were extracted in 01; it is presumed that hygiene problems occurred in the acute phase even in the surrounding area.

In the mid-to long-term phase, 14 subgraphs were indicated in the co-occurrence network diagram as shown in Figure 2b. As shown in the 01 subgraph, the keywords “nursery school”, “kindergarten”, “electricity”, “gas” and “lifeline” are included in large circles. “Dietitian” and “manual” were included in the 04 subgraph. On the contrary, in the medium-to-long term, problems that were seen in the non-disaster period were also extracted, and the 02 subgraph included “allergy”, “cooking”, and “meetup”, and the 08 subgraph included “blood pressure”, “salt” and “fried food”.

## 4. Discussion

This study investigated the health conditions, food, and nutrition issues for mothers and children during the acute to mid-to-long-term phases after the Kumamoto earthquake from objective perspectives by using computer-assisted quantitative text analysis to improve nutrition assistance after a natural disaster. Focus group interviews were conducted with dietitians who provided nutritional assistance for children in the areas affected by the Kumamoto earthquake in 2016. These interviews were about the maternal and child issues during acute- to mid-to-long-term phase in the severely damaged area and surrounding area. From the comparison of the co-occurrence network diagram, there were hygienic problems not only in the acute phase but also in the mid-to-long-term phase in the severely damaged area. Contrastingly, “allergy” was extracted in the surrounding area in the acute and the mid-to-long-term phase, but not extracted as an issue in the acute phase in the severely damaged area. Furthermore, the problems shifted to health and the quality of diet in the mid-to-long-term phase in the surrounding area. In areas with severe damage, it is essential to provide nutrition assistance so that survivors can return to their usual diet as soon as possible.

The purpose of this study was to obtain the overall picture of the health and dietary issues of mothers and children during the Kumamoto earthquake, but new findings were obtained by performing objective analysis by computer using the same data used for qualitative descriptive analysis. One of the new findings was that hygiene problems continued until the mid-to-long-term phase in the severely damaged area. This finding regarding hygiene in the mid-to-long-term phase could not be extracted as a main category when the interview text data from FGI were analyzed using the qualitative descriptive analysis method [8]. The other qualitative analysis of the activity reports of dietitians dispatched to the disaster area also reported that hygiene problems such as food management, drainage, and toilets had occurred, but these problems were at a relatively early stage [13]. There must have been a number of words related to hygiene issues at the FGIs, but other high-impact content may have been the focus of attention. This is considered to be a limit of subjective analysis performed by humans. It has clarified that co-occurrence network analysis can eliminate the subjectivity of the analyst and reduce the disadvantages of qualitative descriptive research conducted by humans; it can be analyzed objectively. On the other hand, from the acute phase to the mid-to-long-term phase, the problem of mother and child shifted to the quality of diet and health, and similar results were obtained both from the subjective analysis by humans and the objective analysis by the computer. However, it was not clear whether the content being spoken was positive or negative just by analyzing the co-occurrence network diagram of nouns by computer analysis. To summarize these new findings, the contents that are easy for humans to assume and the contents that are expected can be analyzed by qualitative descriptive analysis by human, but if the impact was not so large, it was suggested that it could be buried unnoticed. To extract new findings using text mining data, using computers may be able to cover the limits of humans combined with qualitative descriptive analysis and the objective analysis [14].

From the results of this study, it was clarified that the problems after disasters differed in areas where the damage level is different. Especially in the severely damaged areas, hygiene problems had been prolonged. Several reports indicated that the presence or absence of evacuation, which can estimate the scale of damage, is related to health condition. After the Great East Japan Earthquake, survivors who developed obesity were those who evacuated as opposed to those who did not evacuate [15,16,17]. It has been reported that evacuees often develop chronic diseases including cardiovascular diseases [18]. From these reports, it is considered that changes in lifestyle by the damage level have a great influence on the health condition and dietary habits of the disaster area. In the future, it may be necessary to provide long-term assistance by focusing on the areas more devastated by a disaster, rather than simply providing long-term support to the affected areas.

An objective analysis using an automatic computer quantitative analysis revealed that even in areas with severe damage, there were statements about the quality of diet, such as sweets, lunch boxes, and miso soup as the acute phase issues. In the qualitative descriptive analysis by human hand [8], the problems necessary for survival were mainly extracted at the acute phase, so it is thought that the concepts closer to reality were grasped by performing automatic computer quantitative analysis. On the other hand, the problem of allergies has been extracted in the surrounding area from the acute phase, but it was not extracted in the acute phase of the area where the damage was severe. In the severely damaged area, they may have been unable to afford to consider allergies. The problem of allergies was prolonged until the mid-to-long-term phase. However, it is difficult to obtain allergen-free foods during a disaster [4], and it has been reported that about half of allergic children were not able to obtain allergen-free foods for more than a week after the Great East Japan Earthquake [19]. It has been reported that food-allergic patients who were evacuated at emergency shelters as evacuees had difficulty eating the provided meals [3]. Specifically, it has been reported that there were cases of only rice being eaten, and/or cases where allergens were unavoidably eaten [19]. To avoid such hidden dietary problems, survey of the situation in the surrounding areas where the disaster burden is light may be able to identify food problems buried in the severely damaged areas.

One limitation of this study is that the content spoken during the interviews was analyzed in a qualitative survey. Therefore, this was not a quantitative evaluation of the entire area affected by the earthquake. Rather, it is worth noting that the interviewers only listened to singular cases from a small sample size and that the problems mentioned did not necessarily occur throughout the entire area. Additionally, the scope of work and specialty of the interview participants determined which issues were given attention. Therefore, it is possible that the content of the interviews only covered some of the problems that occurred in the disaster area. In the future, by comparing results from this study to epidemiologic surveys, it may be possible to more comprehensively grasp the issues facing mothers and children in disaster areas. Finally, a co-occurrence network can eliminate the subjectivity of the analyst and, by analyzing the data objectively, reduce the typical disadvantages of qualitative research. However, by only using a co-occurrence network diagram that targets nouns, it is not possible to analyze whether the content is spoken in a positive or negative connotation. Therefore, it is only possible to obtain an overview of what was said. Additionally, even when interviewing people with the same title of “registered dietitian” or “dietitian”, the words they used differed greatly depending on details such as where they work or the scope of their work. It was also strongly suggested that participant selection when conducting qualitative research is extremely important.

## 5. Conclusions

This study suggests that child and maternal health and diet, such as hygiene and food allergies, are prolonged problems after disasters. As one of the new possibilities, the possibility of identifying dietary problems by investigating the situation in the surrounding areas was indicated, because it is difficult to investigate in severely damaged areas. In contrast, it is considered that changes in lifestyle by the damage level have a great influence on the health condition and dietary habits of the disaster area. Therefore, it is necessary to provide long-term assistance by focusing on the damage level in the affected areas by disaster, rather than simply providing all-round support to the affected areas.

In the near future, to return to the usual diet of mothers and children as soon as possible after disasters, nutrition assistance should be planned by combining the overall trends obtained in this study with the results of a qualitative descriptive analysis.

## Figures and Tables

**Table 1:** Focus group interview participants.

Group	Participants	Number of Participants	Location of Their Work	Demographic Information
1	Registered Dietician and Dietitians	5	Severely Damaged Area	Local Government Dietitians, Nursery School Dietitian
2	Registered Dietician and Dietitians	8	Surrounding Area	Local Government Dietitians

## Appendix A. Focus Group Interview Script

“Research for improving maternal and child health services for disasters”—Qualitative survey on nutrition-

Focus group interviews script for dietitian.

We will proceed according to the content of the interview script you sent, but if you have any opinions or questions along the way, you can always speak up. Please tell us a lively story unique to group interviews. Then, I will start recording from here.

**1. Before the Disaster**

First of all, please tell us about your position when the disaster occurred, what you had prepared before the disaster, and what kind of goods you had stockpiled, including self-introduction. Please start to say with your number such as I am number one.

Thank you for many stories.

From here, I would like you to freely say “problems” and “how you responded” regarding health, food and nutrition for infant and their mothers to the three phases.

The first phase is immediately after a disaster occurs, and/or relatively early phase.

The second phase is when the turmoil has subsided, a phase that has passed time since the disaster.

Third phase is present phase.

**2. Immediately and Acute Phase after the Disaster**

First of all, please tell us about the problems that children and mothers had in terms of eating habits, nutrition, and health immediately after the disaster, what they had difficulty with, and how they responded.

It doesn’t matter from anyone, so thank you.

**3. After Some Time Has Passed Since the Disaster (in the Mid-to-Long-Term)**

Secondly, please tell us about the problems that children and mothers had in terms of eating habits, nutrition, and health, what they had difficulty with, and how they responded at the second phase.

The second phase is when the turmoil has subsided, a phase that has passed time since the disaster.

**4. The Current**

Third, please tell us about the problems that children and mothers are having now, and the impact that has been affecting them over time, now that three years have passed.

**Other**

Lastly, please tell us what you forgot to say and ideas for improving maternal and child health services in the future.

Thank you so much for all the stories from various angles. This concludes the interview. I’m really thankful to you.
